# Plant Biotechnology

**DOI:** 10.1155/2013/736731

**Published:** 2013-10-10

**Authors:** Khalid Mahmood Khawar, Selma Onarici, Cigdem Alev Ozel, Muhammad Aasim, Allah Bakhsh, Abdul Qayyum Rao

**Affiliations:** ^1^Department of Field Crops, Ankara University, 06110 Ankara, Turkey; ^2^The Scientific and Technical Council of Turkey Genetic Engineering and Biotechnology Institute, Gebze, 41470 Kocaeli, Turkey; ^3^Department of Biology Education, Gazi University, 06500 Ankara, Turkey; ^4^Department of Biology, Karamanoglu Mehmetbey University, 70200 Karaman, Turkey; ^5^Department of Agricultural Sciences, Section Plant Molecular Virology, University of Bologna, 40127 Bologna, Italy; ^6^Centre of Excellence in Molecular Biology (CEMB), University of the Punjab, 87 West Canal Bank Road, Thokar Niaz Baig, Lahore 53700, Pakistan

It is a privilege, pleasure, and honour to present this special issue to the international scientific community. The editorial board is confident that it is a truly good publication; as the papers published in this issue cover a broad spectrum of topics on plant biotechnology, that could be of wide interest. 

This special issue received 20 papers, of which 2 papers were withdrawn and 4 were rejected (not for their quality but for their relatedness to the special issue). 

The guest editorial board of this issue would like to mention that the published papers in this special issue contain one paper on molecular and genetic diversity from China that studied 115 sugarcane genotypes based on five genomic simple sequence repeat marker (gSSR) loci and 88 polymorphic alleles of loci as detected by capillary electrophoresis. The results indicated large intrapopulation genetic variation compared with interpopulation variation. The knowledge obtained in this study should be useful for future breeding programs. 

One paper discusses the problem of mantled fruits as a result of somaclonal variation in oil palm plantlets regenerated via tissue culture. The molecular aspects of the occurrence possibly due to gene repression such as DNA methylation, histone methylation, and histone deacetylation are discussed. This paper described the total protein polymorphism profiles of somaclonal variants of oil palm and the effects of histone deacetylation on this phenomenon.

One paper presents cytogenetic study of Papaver species that introduced a new concept by developing ice cold water instead of bromonaphthalene for chromosome studies.

The papers on plant cell and tissue culture include a review on genetic transformation in citrus; micropropagation of *Origanum acutidens* (Hand.-Mazz.) Ietswaart using stem node explants; use of tissue culture techniques for producing virus-free plant in garlic; adventitious shoot regeneration from leaf explant of aquatic plant *Hygrophila polysperma (Roxb.) T. Anderson)*; secondary somatic embryogenesis in *Hovenia dulcis *Thunb.; effects of IAA, IBA, NAA, and GA3 on rooting and morphological features of *Melissa officinalis* L. stem cuttings; comparison of different methods for separation of haploid embryo induced through irradiated pollen and their economic analysis in melon (*Cucumis melo *var. inodorus); and comparative studies on cellular behaviour of carnation (*Dianthus caryophyllus* Linn. Cv. Grenadin) grown *in vivo* and *in vitro* for early detection of somaclonal variations and the influence of 1-triacontanol on the growth, flowering, and quality of potted *Bougainvillea glabra* var. “Elizabeth Angus” under natural conditions. Still in another paper published in this issue, there is important information about “*Annotation of differentially expressed genes in the somatic embryogenesis of Musa and their location in the banana genome*." The important information about TDFs sequences opens new possibilities for an in-depth study on molecular and biochemical research pertaining to zygotic and somatic embryogenesis in *Musa* species.

These highly esteemed papers are easily available in the form of open access papers at the website of the journal (http://www.hindawi.com/journals/tswj/psi/biotechnology/). The journal is indexed in many important international databases including Science Citation Index Expanded, Zoological Record, and BIOSIS Previews. 

